# Development and Validation of an RNA-Binding Protein-Based Prognostic Model for Ovarian Serous Cystadenocarcinoma

**DOI:** 10.3389/fgene.2020.584624

**Published:** 2020-10-15

**Authors:** Yunan He, Sen Zeng, Shunjie Hu, Fengqian Zhang, Nianchun Shan

**Affiliations:** ^1^Department of Gynecology and Obstetrics, Sun Yat-sen Memorial Hospital, Sun Yat-sen University, Guangzhou, China; ^2^Department of Neurology, The Third Xiangya Hospital of Central South University, Changsha, China; ^3^Zhongshan School of Medicine, Sun Yat-sen University, Guangzhou, China; ^4^Department of Gynecology and Obstetrics, Xiangya Hospital, Central South University, Changsha, China

**Keywords:** ovarian serous cystadenocarcinoma, RNA-binding proteins, prognostic model, overall survival, bioinformatics

## Abstract

Ribonucleic acid-binding proteins (RBPs) are reportedly involved in tumor progression and recurrence; however, the functions and mechanisms of action of RBPs in ovarian serous cystadenocarcinoma (OSC) are not known. To address these issues, gene expression profiles of OSC tissues from The Cancer Genome Atlas (TCGA) and normal tissues from the Genotype-Tissue Expression database were compared in order to identify RBPs that are differentially expressed in OSC. We also analyzed the biological functions of these RBPs and their relationship to clinical outcome. There were 190 RBPs that were differentially expressed between OSC and normal tissues, including 93 that were upregulated and 97 that were downregulated. Five of the RBPs were used to construct a prediction model that was evaluated by univariate and multivariate Cox regression analyses. TCGA data were randomly divided into training and test cohorts, and further categorized into high- and low-risk groups according to risk score in the model. The overall survival (OS) of the high-risk group was shorter than that of the low-risk group (training cohort *P* = 0.0007596; test cohort *P* = 0.002219). The area under the receiver operating characteristic curve of the training and test cohorts was 0.701 and 0.638, respectively, demonstrating that the model had good predictive power. A nomogram was established to quantitatively describe the relationship between the five prognostic RBPs and OS in OSC, which can be useful for developing individualized management strategies for patients.

## Introduction

Ovarian cancer, a common gynecologic cancer, accounts for just 3% of newly diagnosed tumors but is the fifth leading cause of cancer-related deaths in women; this is partly attributable to the difficulty of early diagnosis and high rates of metastasis and recurrence (Li et al., 2012; Xiong et al., 2018). Ovarian serous cystadenocarcinoma (OSC) is the most common subtype of ovarian cancer (60%–80% of ovarian epithelial tumors) (Li et al., 2012; Kaldawy et al., 2016). In most cases, OSC is detected at an advanced stage and recurrence after treatment is common (Torre et al., 2017). There is therefore a need to clarify the molecular mechanisms underlying OSC pathogenesis and progression so that more effective therapeutic strategies can be developed.

Ribonucleic acid (RNA)-binding proteins (RBPs) participate in the formation of the ribonucleoprotein (RNP) complex for protein synthesis (Dreyfuss et al., 2002). Over 1500 RBPs have been identified to date (Gerstberger et al., 2014) and play a critical role in RNA processing by regulating mRNA stability, localization, alternative splicing, polyadenylation, and translation efficiency (Brinegar and Cooper, 2016; Protter and Parker, 2016; Masuda and Kuwano, 2019). Dysregulation of RBP expression has been implicated in numerous human diseases (Brinegar and Cooper, 2016; Newman et al., 2016). For example, mutations in the genes encoding the RBPs Fused in sarcoma (FUS) and TAR DNA-binding protein 43 (TDP-43) have been linked to the pathogenesis of amyotrophic lateral sclerosis, and the proteins were depleted from the nucleus and aggregated in the cytoplasm in affected neurons (Brinegar and Cooper, 2016). The RBPs Elav-like family (CELF) and Muscleblind-like (MBNL) contribute to the pathogenesis of myotonic dystrophy by reverting to fetal expression patterns and promoting fetal mRNA processing in adult tissues (Brinegar and Cooper, 2016).

RBPs are also associated with cancer development, as dysregulation of RBP expression alters the expression of oncogenes and tumor suppression genes (Pereira et al., 2017). Musashi 1 (MSI1) and MSI2 have been shown to increase the levels of Myc and estrogen receptor α1 (ESR1) oncogenes and reduce that of phosphatase and tensin homlog (PTEN) by modulating mRNA stability and protein translation, leading to various types of cancer (Kudinov et al., 2017). LIN-28 homolog B (LIN28B) promotes pluripotency and plays a critical role in colorectal carcinogenesis by interacting with microRNAs of the let-7 family (King et al., 2011; Balzeau et al., 2017). Quaking (QKI), a splicing factor that regulates cell proliferation, is downregulated in lung cancer, which is associated with poor survival (Zong et al., 2014). RNA-binding motif protein 10 (RBM10) is a regulator of alternative splicing in lung adenocarcinoma (Hernandez et al., 2016); and human antigen R (HuR) promotes cell dedifferentiation and proliferation by regulating the stability of target mRNAs in hepatocellular carcinoma (Fernandez-Ramos and Martinez-Chantar, 2015). However, the mechanisms by which most RBPs contribute to carcinogenesis remain unknown.

The aim of the present study was to clarify the role of RBPs in the pathogenesis of OSC. We retrieved RNA sequencing and clinicopathologic data for OSC from The Cancer Genome Atlas (TCGA) database and screened for differentially expressed RBPs. A functional analysis was also carried out in order to identify key RBPs in OSC that can potentially serve as prognostic biomarkers.

## Materials and Methods

### Data Processing

Ribonucleic acid profiles of tumor tissue from OSC patients and normal tissues were obtained from TCGA database. For comparison, we obtained RNA sequences of normal ovarian tissue from the Genotype-Tissue Expression (GTEx) database. RBPs that were differentially expressed between tumor and normal tissues were screened using R v4.0.2 software (The R Project, Vienna, Austria).

### Kyoto Encyclopedia of Genes and Genomes Pathway and Gene Ontology Analyses

To determine the biological function of differentially expressed RBPs, we used the R software packages clusterProfiler, org.Hs.eg.db, enrichplot, and ggplot2 to carry out KEGG and GO analyses, which included cellular component (CC), molecular function (MF), and biological process (BP) as functional domains. A q value or false discovery rate < 0.05 was taken as statistically significant.

### Protein–Protein Interaction Network Construction

Search Tool for the Retrieval of Interacting Genes/Proteins (STRING) was used to investigate the interactions of RBPs. A PPI network and visual subnetwork were constructed using Cytoscape v3.8.0 software (https://cytoscape.org/index.html). Functionally significant RBPs were identified using the Molecular Complex Detection (MCODE) algorithm. RBPs with MCODE score and node counts > 3 were deemed significant, and *P*-values < 0.05 were considered statistically significant.

### Prognostic Model Construction and Validation

The survival package of R software was used for univariate Cox regression analysis of key RBPs; candidate RBPs were selected with the log-rank test and incorporated into a multivariate Cox regression model. The risk score was calculated according to the following formula: risk score = β1 × Exp_1_ + β2 × Exp_2_ + … + βi × Exp_*i*_. We used R software to construct a nomogram to predict overall survival (OS) of OSC patients. The model was validated using data from TCGA database, which were randomly divided into training and test cohorts. With the median risk score as the cutoff, OSC patients were divided into high- and low-risk groups, and the log-rank test was used to compare differences in OS between them. *P* < 0.05 was considered statistically significant. Receiver operating characteristic (ROC) curve analysis was also performed to evaluate the predictive value of the model, which was validated using data from the Human Protein Atlas (HPA) database.

## Results

### Identification of Differentially Expressed RBPs in OSC Patients

**FIGURE 1:**
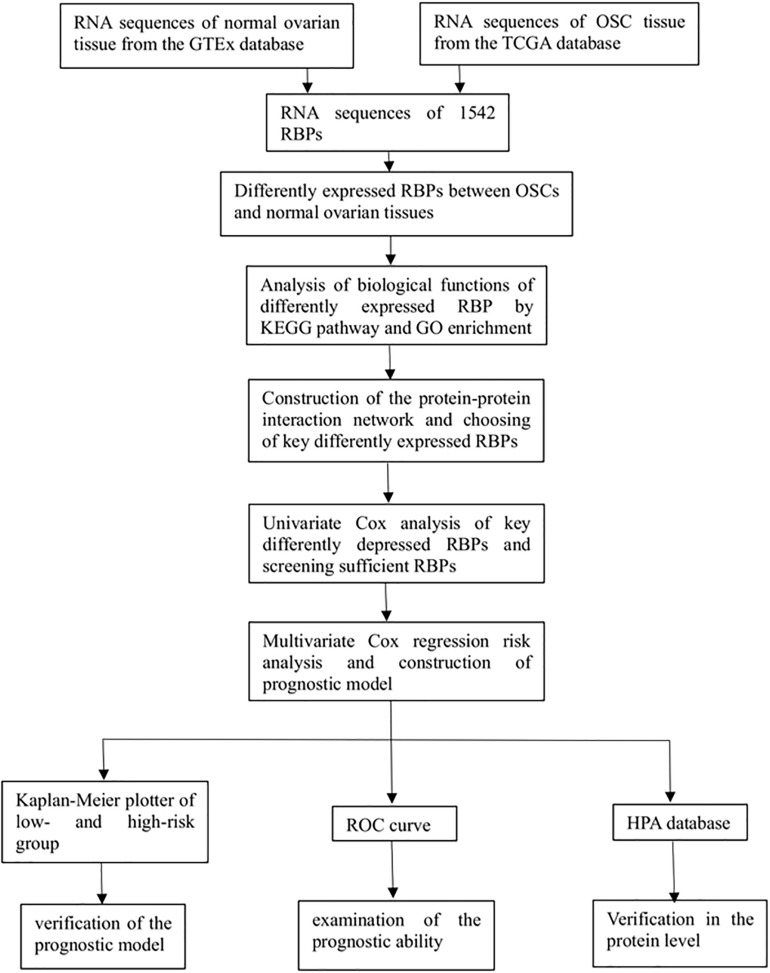
Flow diagram of this study.

**FIGURE 2:**
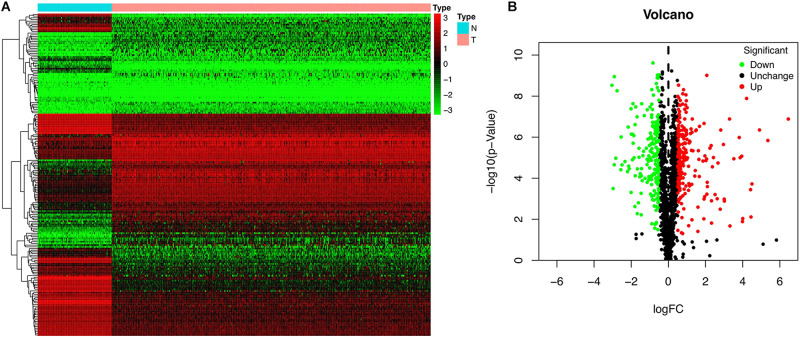
Differentially expressed RBPs in OSC. **(A)** Heat map. **(B)** Volcano plot.

We investigated the functions and prognostic value of RBPs in OSC patients; the flow diagram of the study is shown in Figure 1. We downloaded RNA sequences of 379 OSC patients from TCGA database; 88 normal ovarian tissue samples obtained from the GTEx database were used as a control. The RNA sequences of 1542 RBPs (Gerstberger et al., 2014) were ultimately included in the analysis; 190 sequences encoded RBPs that were differentially expressed between normal and tumor tissues (P < 0.05, | log2 fold change| > 1.0), including 93 upregulated and 97 downregulated RBPs (Figure 2). All up- or down-regulated RBP genes in OSC has been listed in the Supplementary files.

### KEGG Pathway Enrichment and GO Analyses of Differentially Expressed RBPs

**FIGURE 3:**
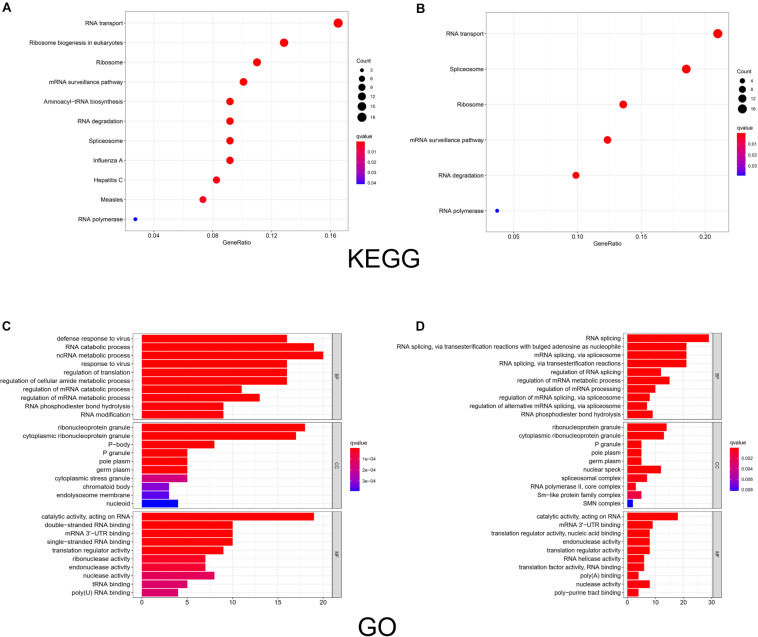
KEGG pathway analysis and GO enrichment analysis of differentially expressed RBPs. The size of a dot in the KEGG analysis represents the relative abundance of the corresponding RBP. The color represents the *q* value, with a darker red color indicating a smaller *q* value. And the color bar in the legend of GO analysis represents the q value of the corresponding item. BP, biological process; CC, cellular component; MF, molecular function. **(A)** KEGG analysis of upregulated RBPs. **(B)** KEGG analysis of downregulated RBPs. **(C)** GO enrichment analysis of upregulated RBPs. **(D)** GO enrichment analysis of downregulated RBPs.

We used R software to evaluate the enrichment of the identified RBP-encoding genes under biological processes, metabolic mechanisms, and molecular functions. The results of the KEGG analysis showed that the upregulated RBPs were significantly enriched in pathways related to RNA transport, ribosome biogenesis in eukaryotes, and ribosome (Figure 3A), whereas downregulated RBPs were enriched in RNA transport, spliceosome, and ribosome (Figure 3B). GO analysis revealed that under BP, upregulated RBPs were mainly involved in defense response to virus, RNA catabolic process, and non-coding RNA metabolic process. Meanwhile, downregulated RBPs were involved in RNA splicing; RNA splicing, via transesterification reactions with bulged adenosine as nucleophile; mRNA splicing; and mRNA splicing via spliceosome. Under CC, both upregulated and downregulated RBPs were enriched in RNP granule, cytoplasmic RNP granule, and P-body. Under MF, both upregulated and downregulated RBPs were enriched in catalytic activity, acting on RNA, and mRNA 3’-untranslated region (UTR) binding; upregulated RBPs were also enriched in double-stranded RNA binding (Figure 3C), and downregulated RBPs were enriched in translation regulator activity and nucleic acid binding (Figure 3D).

### PPI Network Construction and Key Module Selection

**FIGURE 4:**
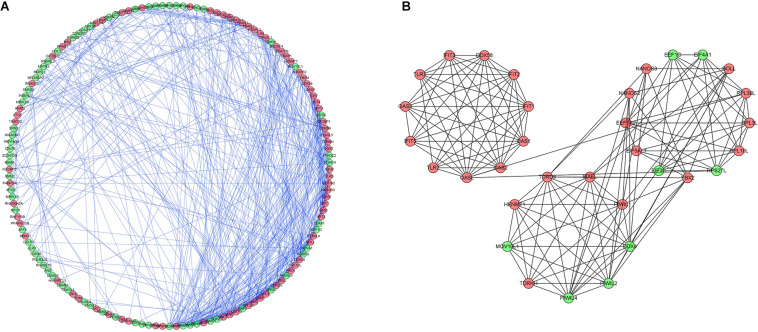
Protein-protein interaction network and modules analysis. **(A)** Protein–protein interaction network of differentially expressed RBPs. **(B)** Three critical modules from PPI network. Green circles: downregulation with a fold change of more than 2. Red circles: upregulation with fold change of more than 2.

To investigate the interactions of differentially expressed RBPs and identify key RBPs related to OSC, we constructed a PPI network using data from the STRING database and Cytoscape software. The PPI network included 190 nodes and 493 edges. A coexpression network was constructed using the MCODE tool and the top 3 modules and genes were selected and visualized according to their risk scores (Figure 4). The RBPs in the key modules were associated with the defense response to virus, translation, and RNA binding.

### Construction of a RBP-Based Prediction Model for OSC

**FIGURE 5:**
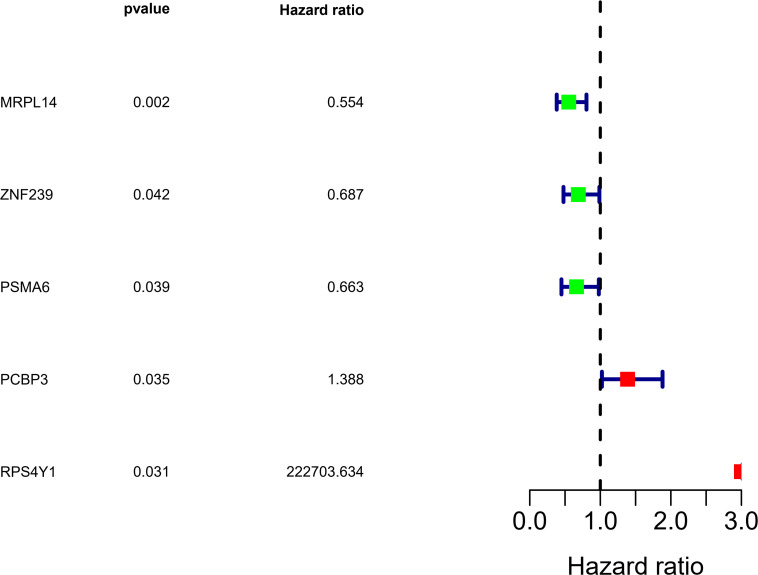
Identification of prognosis-related RBPs by multivariate Cox regression analysis.

**FIGURE 6:**
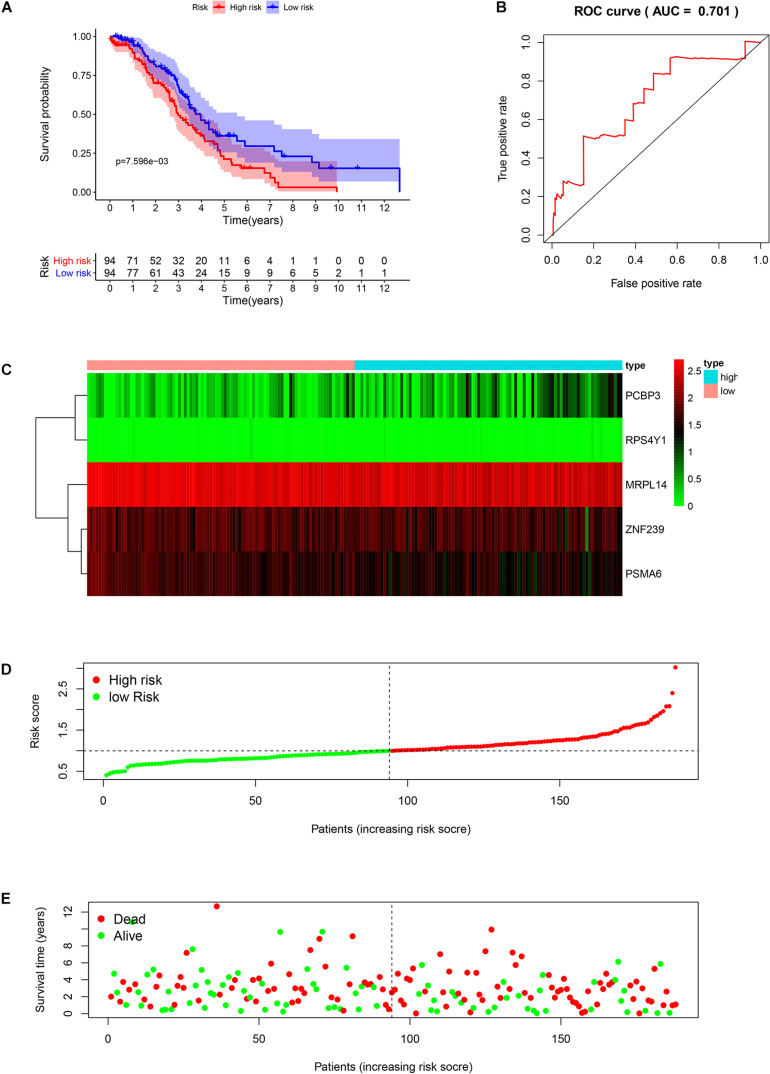
Risk score analysis of the training cohort in TCGA using the 5-gene prognostic model. **(A)** Survival curve for low- and high-risk subgroups. **(B)** ROC curve for predicting OS based on risk score. **(C)** Expression heat map. **(D)** Risk score distribution. Patients were assigned to the training group based on risk score for determination of median risk score. **(E)** Survival status. The dashed line represents the median risk score; most patients on the right side had died, revealing a trend of greater risk of death with increasing risk score.

**FIGURE 7:**
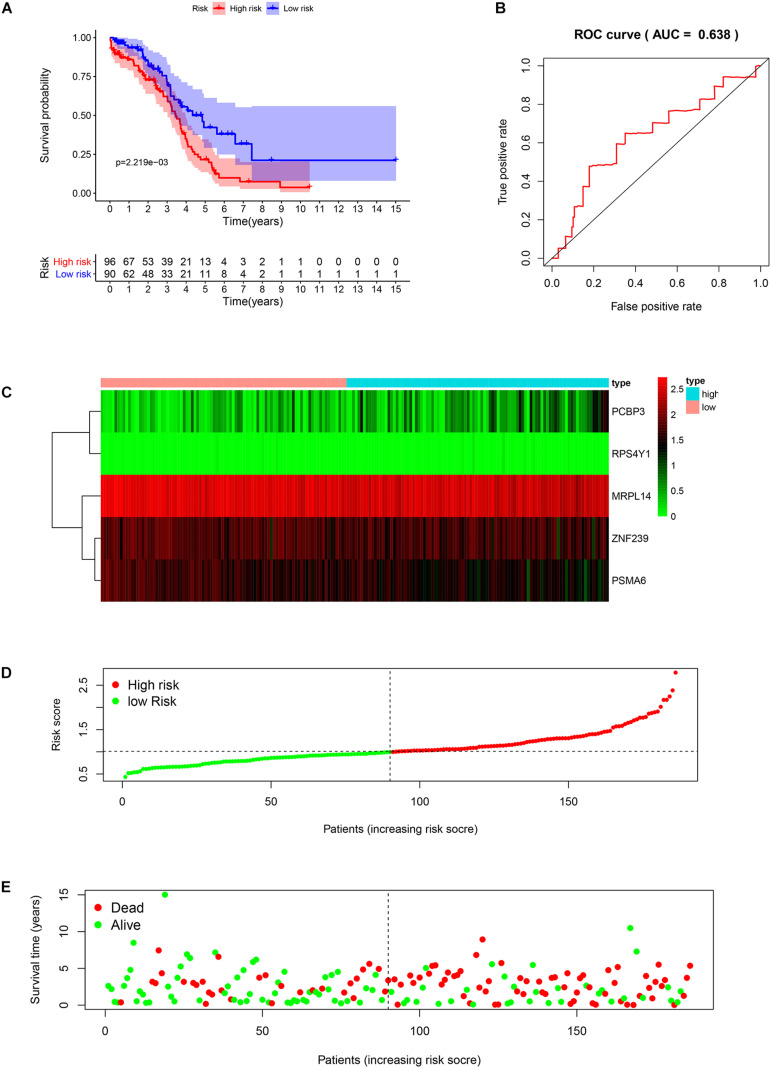
Risk score analysis of the test cohort in TCGA using the 5-gene prognostic model. **(A)** Survival curve for low- and high-risk subgroups. **(B)** ROC curve for predicting OS based on risk score. **(C)** Expression heat map. **(D)** Risk score distribution. Patients were assigned to the training group based on risk score for determination of median risk score. **(E)** Survival status. The dashed line represents the median risk score; most patients on the right side had died, revealing a trend of greater risk of death with increasing risk score.

We analyzed the RNA sequences of 9 RBPs selected from the PPI network and evaluated their clinical and prognostic significance. The results of the univariate Cox regression analysis revealed that five of the RBPs (mitochondrial ribosomal protein L14 [MRPL14], zinc finger protein 239 [ZNF239], proteasome 20S subunit α6 [PSMA6], poly[RC]-binding protein 3 [PCBP3], and ribosomal protein S4 Y-linked 1 [RPS4Y1]) were related to prognosis in OSC. To further assess their influence on OS, we performed a multivariate Cox regression analysis and found that the five RBPs were independent predictors of OS in OSC patients (Figure 5). We constructed a prediction model by calculating the risk score for each patient using the following formula: risk score = (−0.34749 × Exp[MRPL14]) + (−0.17478 × Exp[ZNF2 39]) + (−0.47382 × Exp[PSMA6]) + (0.41487 × Exp[PCBP3]) + (3.46278 × Exp[RPS4Y1]). A total of 379 OSC patients in TCGA were randomly divided into training and test cohorts and further classified into low- and high-risk subgroups according to median risk score. To evaluate the predictive value of our model, we performed a survival analysis of the cases. In both the training and test cohorts, the high-risk group had shorter OS than the low-risk group (training cohort *P* = 0.0007596, test cohort *P* = 0.002219) (Figures 6A, 7A). The heatmap of RBP expression, survival status, and risk scores of the low- and high-risk subgroups of the training and test cohorts are shown in Figures 6C–E, 7C–E. The time-dependent ROC curve analysis showed that the area under the ROC curve of the RBP-based risk score model was 0.701 and 0.638 for the training and test cohorts, respectively (Figures 6B, 7B), indicating a moderate predictive power.

### Construction of a Nomogram Based on RBPs

**FIGURE 8:**
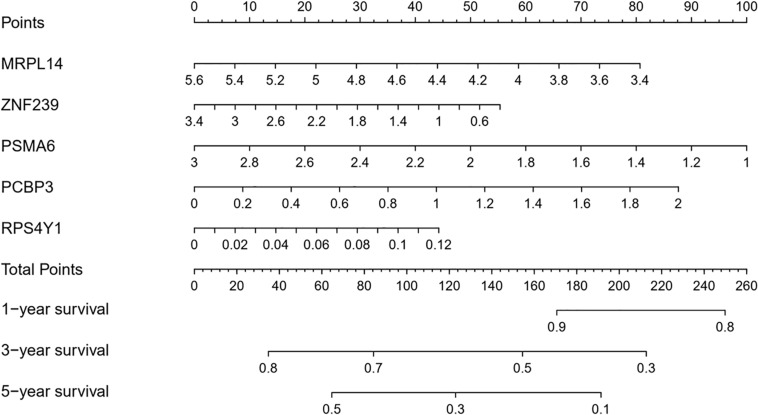
Nomogram for predicting 1-year, 3-year, and 5-year OS of OSC patients in TCGA database.

A nomogram was constructed to quantitatively assess the role of the five RBPs in the prediction model for OSC patient survival (Figure 8). Based on the multivariate Cox analysis, we assigned scores of each variable to the scale of the nomogram, determined the score of each variable, and calculated the total scores of the five RBPs for each patient. The total score was normalized to a distribution ranging from 0 to 100 and used to calculate the 1-year, 3-year, and 5-year estimated OS rates of OSC patients.

**FIGURE 9:**
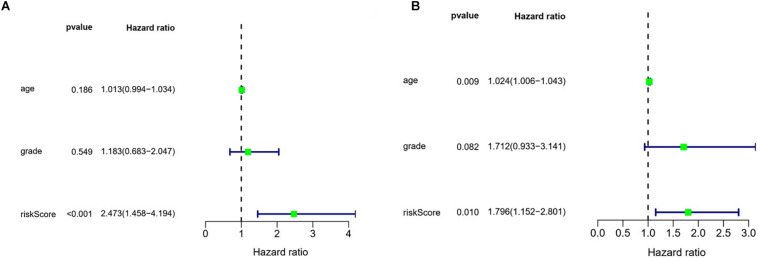
Evaluation of the prognostic value of different clinical parameters by univariate analysis. **(A)** Training cohort. **(B)** Test cohort.

**FIGURE 10:**
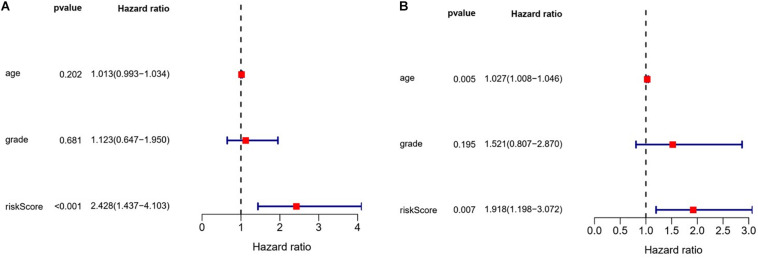
Evaluation of the prognostic value of different clinical parameters by multivariate analysis. **(A)** Training cohort. **(B)** Test cohort.

We also evaluated the prognostic significance of various clinical characteristics of OSC patients in TCGA by Cox regression analysis. The univariate analysis showed that risk scores were independent risk factors for OS (training cohort *P* < 0.001, test cohort *P* = 0.010), while age and tumor grade were unrelated to OS (Figure 9). The multivariate regression analysis showed that risk scores were independent prognostic factors for OS in OSC patients (training cohort *P* < 0.001, test cohort *P* = 0.007) (Figure 10).

### Expression of Prognostic RBPs

**FIGURE 11:**
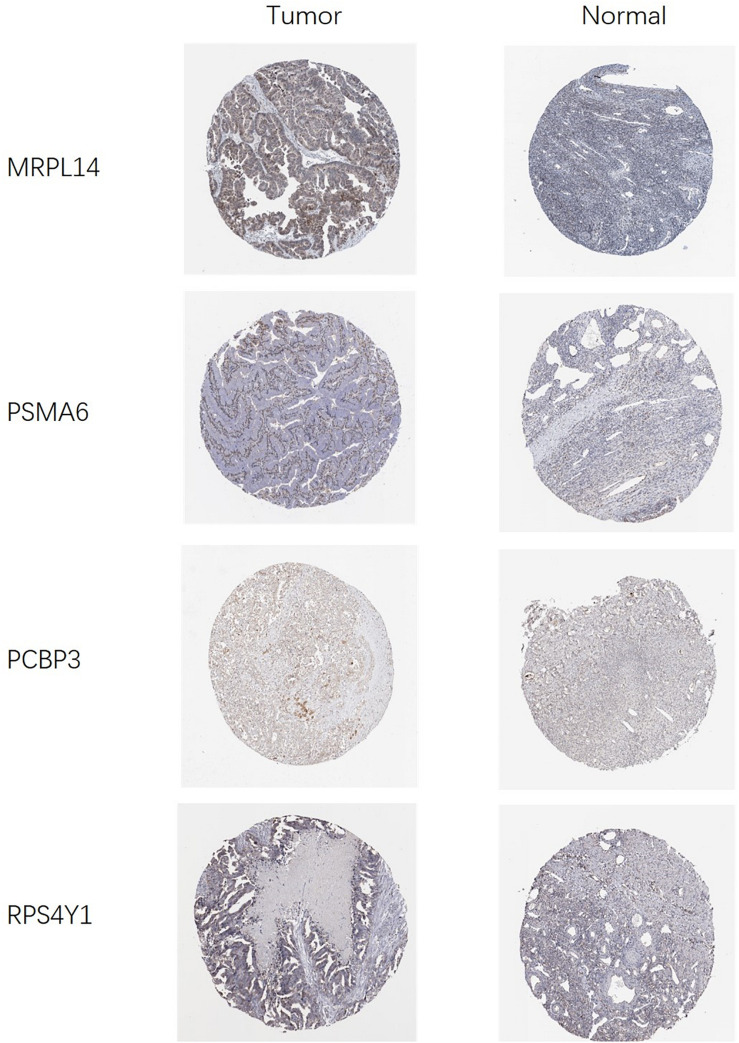
Validation of prognostic RBP expression in OSC and normal ovarian tissue using the HPA database.

In order to clarify the expression of the five prognostic RBPs in OSC patients, we examined immunohistochemistry data from the HPA database. MRPL14 was highly expressed in OSC tissue compared to normal tissue. In contrast, the immunoreactivity of PSMA6, PCBP3, and RPS4Y1 in OSC tissue was relatively low (Figure 11). ZNF239 protein expression data were not available in the HPA.

## Discussion

Only a small fraction of RBPs have been identified as being related to tumor recurrence and progression, and in most cases the mechanism of action has not been reported. Bioinformatics approaches allow investigation of the diagnostic or prognostic significance of changes in RBP expression. Our study identified 190 RBPs that were differentially expressed between OSC and normal tissues. Five of the RBPs were used to construct a risk prediction model, which showed moderate performance in predicting OSC patient survival.

The results of the GO and KEGG pathway analyses revealed that the differentially expressed RBPs were significantly enriched in the defense response to virus as well as RNA- and protein translation-related processes that have been linked to the pathogenesis of various human diseases (Scotti and Swanson, 2016; Anastasiadou et al., 2018; Grafanaki et al., 2019). RBPs form the RNP complex that regulates RNA stability and hence, gene expression; dysfunction of the RNP complex can lead to cancer development and progression (Carotenuto et al., 2019). The RBP ribonucleoprotein 1, translational regulator 1 (LARP1) promotes ovarian cancer progression and by altering the stability of its target mRNAs B cell lymphoma 2 (BCL2) and BCL-2–interacting killer (BIK) (Hopkins et al., 2016). LIN28B inhibits the apoptosis of ovarian cancer cells and promotes cancer progression by binding to AKT2 mRNA and increasing the expression of the protein (Lin et al., 2018).

The PPI of the differentially expressed RBPs identified in this study reveals an important role for these proteins in tumorigenesis and cancer progression. Eukaryotic translation elongation factor 1 α2 (EEF1A2) is an oncogene that promotes ovarian carcinogenesis and inhibits apoptosis of ovarian cancer cells (Lee, 2003). Toll-like receptor 3 (TLR3) was shown to play a dual role in ovarian cancer by eliminating tumor cells via upregulation of interferons and activation of natural killer cells and also by promoting cancer development (Husseinzadeh and Davenport, 2014).

Five of the differentially expressed RBPs, namely MRPL14, ZNF239, PSMA6, PCBP3, and RPS4Y1, showed prognostic value in OSC by univariate and multivariate Cox regression analyses. MPRL14 was found to be upregulated in tumor cells and its expression was positively correlated with the outcome of OSC patients. Reduced ZNF239 and PSMA6 and elevated PCBP3 and RPS4Y1 levels were associated with worse prognosis. The mitochondrial ribosomal proteins (MRPs) are the counterpart of cytoplasmic ribosomes relating to maintain mitochondrial DNA stability (O’Brien et al., 1999). The MRPL14 single nucleotide polymorphism may be related to diabetic retinopathy through steroid metabolism or insulin resistance (Lin et al., 2013). MRPL14 is highly expressed in thyroid tumor (Jacques et al., 2013), but does not reveal the relationship with prognosis. In the past 5 years, no relationship between ZNF239 and any type of tumor has been reported. The proteasome gene, PMSA6, encodes the a1 protein, which is involved in the formation of the outer rings of the 20s core proteasome and is subject to post-translational regulation (Choudhary et al., 2009; Wang et al., 2013). The location of the PSMA6 gene occurs in a region containing microsatellites that have been implicated in coronary artery disease (CAD) (Alsmadi et al., 2009), type 2 diabetes mellitus (T2DM) (Sjakste et al., 2007), Grave’s disease (Sjakste et al., 2004), asthma (Zemeckiene et al., 2015), ankylosing spondylitis (Zhao et al., 2015), and myocardial infarction (Liu et al., 2009). In a lung cancer study, the expression of PSMA6 was up-regulated, and knocking out PSMA6 could induce lung cancer tumor cell apoptosis or the cell cycle to enter the arrest phase (Kakumu et al., 2017). However, in our study, the expression of PSMA6 in OSC is down-regulated, and the low expression of PSMA6 is associated with a worse OSC prognosis, which may be due to the different effects of PSMA6 expression on proteasome activity. PSMA6 has carcinogenic effects in various tissue tumors. Actually, the ubiquitination-proteasome degradation pathway has been proved to be the key to cell survival and proliferation. Therefore, the detailed molecular mechanism of PSMA6 in OSC needs to be revealed. The poly(C) binding proteins (PCBPs), an RNA-binding protein involved in post-transcriptional regulation, whose important functions are mRNA activation, translation activation and translation silencing (Makeyev and Liebhaber, 2002). A study of pancreatic ductal carcinoma showed that the content of PCBP3 protein in postoperative tissues was significantly related to the survival time of patients, and the prognosis of the group with lower PCBP3 protein content was worse (Ger et al., 2018). This is consistent with the results of our study. Otherwise, the initiation of RPS4Y1 expression is the basis of Y chromosome activation (Zhou et al., 2019). There is currently no report on the relationship between RPS4Y1 and tumors. However, studies about these five RBP genes in ovarian cancer are rarely seen and the molecular link between these five RBPs and OSC progression has yet to be elucidated. Clinical specimen validation and follow up data of OSC patients are also wanted in the following research. The results of the ROC curve analysis indicated that the five RBPs showed moderate performance in identifying OSC patients who are at risk of progression; the nomogram model constructed to predict 1-year, 3-year, and 5-year OS in OSC patients yielded similar findings.

This study had some limitations. Firstly, the prediction model was based on TCGA data and no clinical validation or prospective clinical study was conducted; moreover, the limited clinical information in the TCGA dataset may have diminished the reliability of the Cox regression analysis. Nonetheless, our model based on five RBPs showed great potential being used to predict OSC patient prognosis, which can inform clinical decisions and lead to better outcomes.

## Data Availability Statement

Publicly available datasets were analyzed in this study; these data can be found here: The Cancer Genome Atlas (https://portal.gdc.cancer.gov/).

## Author Contributions

YH and NS designed the study. SZ and YH performed the experiments. YH and SH analyzed the data. YH, SH, and FZ wrote the manuscript. All authors reviewed the final version of the article.

## Conflict of Interest

The authors declare that the research was conducted in the absence of any commercial or financial relationships that could be construed as a potential conflict of interest.
